# Myeloid-Derived Suppressor Cells in Patients With Acute Pancreatitis With Increased Inhibitory Function

**DOI:** 10.3389/fimmu.2022.840620

**Published:** 2022-07-14

**Authors:** Lili Ding, Minjie Wan, Dong Wang, Huiru Cao, Haijiao Wang, Pujun Gao

**Affiliations:** ^1^ Intensive Care Unit, The First Hospital of Jilin University, Changchun, China; ^2^ Department of Medical Ultrasonics, The First Affiliated Hospital of Sun Yat-Sen University, Guangzhou, China; ^3^ Department of Gastroenterology, Shengjing Hospital of China Medical University, Shenyang, China; ^4^ Department of Gynecology Oncology, The First Hospital of Jilin University, Changchun, China; ^5^ Department of Hepatology, The First Hospital of Jilin University, Changchun, China

**Keywords:** MDSC, immunosuppression, arginase-1, ROS, acute pancreatitis

## Abstract

Acute pancreatitis (AP) is pancreatic or systemic inflammation without or with motion organ dysfunction. Severe acute pancreatitis (SAP) is the main cause of death for patients with AP. A pro-/anti-inflammatory imbalance is considered the key regulation of disease severity. However, the real mechanism of SAP remains unclear. This study aimed to identify the frequency and specific roll of myeloid-derived suppressor cell (MDSC) in AP. We evaluated MDSC frequency and disease severity by analyzing MDSCs in the peripheral blood of healthy controls (HCs) and patients with mild acute pancreatitis (MAP) and SAP by flow cytometry. We also compared the frequency and inhibitory ability of MDSCs from HCs and SAP, and finally detected the reason for the difference in inhibitory ability. AP was marked by expansion of MDSCs as well as its subsets, granulocytic MDSCs (G-MDSCs) and monocytic MDSCs (M-MDSCs). The proportion of MDSC in the peripheral blood mononuclear cells of patients with AP was increased and positively correlated with AP severity. The frequency of MDSC was decreased after treatment compared with pre-treatment. CD3+ T cells were remarkably inhibited by MDSC derived from the patients with SAP. In the expression of arginase-1 (Arg-1) and reactive oxygen species (ROS), the MDSCs from patients with SAP increased. These findings demonstrated that MDSCs expanded in the peripheral blood in patients with AP, especially in those with SAP. Moreover, the inhibitory ability of MDSCs was increased in the patients with SAP compared with that in the HCs. The enhanced suppressive function was possibly caused by an overexpression of Arg-1 and ROS.

## 1 Introduction

Acute pancreatitis (AP) is an inflammatory disease of the pancreas caused by tissue injury or necrosis due to pancreatic enzymes. According to disease severity, AP is categorized as either mild acute pancreatitis (MAP), moderate–severe acute pancreatitis (MSAP), or severe acute pancreatitis (SAP) (1). SAP is defined with local or remote organ dysfunction (2), accounting for the majority of the death rate. Damaged pancreatic acinar cells induce a systemic inflammatory response syndrome (SIRS) by releasing cytokines, chemokines, and immune cells, such as such as IL-1β, IL-6, Th1, and Th17 (3–5). Compensatory anti-inflammatory syndrome (CARS) is a regulatory procedure limiting the excessive inflammatory response, involving regulatory immune cells, such as regulatory T cells (Tregs) and type 2 macrophages (M2) (6, 7). The balance between SIRS and CARS determines the systemic immune status.

Myeloid-derived suppressor cells (MDSCs) are a group of heterogeneous immature cells in the bone marrow (8). Immature myeloid cells originate from the bone marrow and quickly differentiate into mature granulocytes, macrophages, or dendritic cells physiologically. However, the differentiation of immature myeloid cells into mature myeloid cells is blocked by specific pathological conditions, such as cancer, infectious diseases, trauma, or some autoimmune disorders. In the human body, the phenotype of MDSCs is CD11b+ HLA-DR- and according to phenotypic and morphological features. MDSCs can be classified into two main subsets: CD14+ monocytic MDSCs (M-MDSCs) and CD66b+ granulocytic MDSCs (G-MDSCs) (9). Functionally, MDSCs inhibit both innate and adaptive immune responses by secreting arginase 1 (Arg-1), inducible nitric oxide synthase (iNOS), reactive oxygen species (ROS), and peroxynitrite (8). MDSCs suppress lymphocyte proliferation or T cell response to control tumor-associated inflammation, inflammation of autoimmune diseases and other inflammatory diseases, such as hepatic steatosis and sepsis (10–12). MDSCs infiltrated the pancreas in an AP mice model; however, the frequency of MDSCs in patients with AP of different stages and the exact role of MDSCs remain unclear (13). The findings have indicated the potential function of MDSCs in the pathogenesis of AP, providing a new therapeutic target.

This study aimed to identify the frequency of MDSCs in peripheral blood mononuclear cells (PBMCs) and its correlation with AP severity, to explore the immune status concerning MDSCs in AP, and to provide potential therapeutic strategies for SAP cases.

## 2 Materials and Methods

### 2.1 Study Design

The study included 22 patients with MAP admitted to the Department of Hepatology and 25 patients with SAP in the Intensive Care Unit of the First Hospital of Jilin University from November 2018 to June 2019, and 26 healthy volunteers who were recruited as health controls (HCs) from the Physical Examination Center in the First Hospital of Jilin University (Changchun, Jilin, China) during the same period. All the patients with AP were diagnosed and graded according to the Atlanta criteria (14). HCs were matched with patients with AP for sex, age, ethnicity without any diseases including acute or chronic inflammation, tumors, and allergies. The exclusion criteria were (1): history of use of anti-inflammatory drugs, including corticosteroids and non-steroidal anti-inflammatory drugs within 30 days before enrollment and immune-suppressive drugs within 3 months (2); with history of autoimmune disease, tumors, inflammatory diseases, or other chronic diseases (3); pregnancy; and (4) age < 18 years. The experimental protocol was established according to the Declaration of Helsinki guidelines and was approved by the Human Ethics Committee of Jilin University (approval number: 219-323).

### 2.2 Treatment and Clinical Examination

Patients with MAP and SAP were treated with conventional treatment, such as diet resistance, administration of pain relievers, and inhibiting gastric acid and pancreatin secretion. In addition, closed monitoring, fluid resuscitation, appropriate antibiotic treatment, mechanical ventilation, renal replacement therapy, and nutritional support were implemented in the SAP cases.

Each patient’s clinical data including age, sex, and biochemical laboratory test information was collected from the hospital records. The peripheral blood cell counts were analyzed using scatter turbidimetry using a Siemens special protein analyzer (Siemens Healthcare Diagnostics Products, GmbH, Munich, Germany), and the concentrations of plasma CRP, amylase (AMY), and serum calcium concentration were tested using an ADVIA 1650 biochemical analyzer (Bayer, Pittsburg, PA, USA).

### 2.3 Isolation of PBMCs

Venous blood was collected from the HCs and patients with MAP and SAP. Ascitic fluid was taken from patients with SAP who had drainage. PBMCs and ascitic fluid mononuclear cells (ASMCs) were isolated by density-gradient centrifugation from the venous blood and ascitic fluids, respectively. Propidium iodide (PI) was used to discriminate dead cells. The following anti-human fluorescence conjugated antibodies and related isotype controls were used: anti-human CD11b-APC (clone ICRF44, Biolegend), anti-human HLA-DR-APC-Cy7 (clone L243, Biolegend), anti-human CD66b-Percpcy5.5 (clone G10F5, Biolegend), anti-human CD14-FITC (clone M5E2, Biolegend), Arg-1-PE (clone 14D2C43, Biolegend), anti-human CD155-BV421 (clone SKII.4, BD), and PI (Solarbio).

### 2.4 Flow Cytometry

For the MDSC analysis, surface staining was performed as follows. PBMCs (2-5×10^5^/tube) were stained in duplicate with APC-anti-CD11b, APC-Cy7-anti-HLA-DR, PerCP-Cy5.5-anti-CD66b, and FITC/BV510-anti-CD14 in the dark at 4°C for 30 min. Negative controls were stained with isotype-matched control antibodies. PI was used to identify the live cells for surface staining. The stained cells were analyzed by flow cytometry. For intracellular staining, the cells were differentiated dead cells from live cells with Fixable Viability Dye (FVD, eBioscience) labeled with BV510, and fixed and permeabilized using a fixation/permeabilization kit (BD Biosciences) 200 µL/tube at 4°C in the dark for 30 min. After washing cells with buffer, intracellular antibody Arg-1 was added for staining. The ROS probe (BestBio, BB-4705) was performed in a diluted solution as recommendation. PBMC was stained with ROS probe in 37°C for 20min following surface staining, mixed completely every 5 mins, washed with PBS for 3 times, and then detected with flow cytometer under FITC panel.

### 2.5 Cell Isolation

CD3+ T cells were isolated using the CD3+ T Cell Isolation Kit (Miltenyi Biotec) from the peripheral blood of the HCs and the patients with SAP. The obtained PBMCs were counted by staining with Trypan blue and the human CD3+ T Cell Microbead antibody at 4°C for 15 min in the dark. A 50-μL and 20-μL anti-biotin microbeads per 10^7^ cells were added. Labelled PBMCs were resuspended by buffer and isolated by LS (or MS) column *via* positive screening. The number and purity of the sorted cells were determined by blood cell counting and flow cytometry, respectively.

The PBMCs obtained from SAP patients and HCs were added with anti-human APC-anti-CD11b and APC/CY7-anti-HLA-DR (1 µL/10^6^ cells), and incubated for 30 min at 4°C in the dark for staining. The cells were washed and resuspended with buffer. To remove dead cells, 10 µL of diluted PI was added. CD11b+ HLA-DR- cells were sorted by FACS Aria II (Becton, Dickinson and Company, Franklin Lakes, NJ, United States), and collected in complete medium of RPMI-1640. The purity of MDSC was determined by flow cytometry after separation.

### 2.6 Proliferation Assays

Carboxyfluorescein succinimidylester (CFSE)-labeled CD3+ T cells (5×10^4^) were seeded with or without MDSCs (5×10^4^) in 96 well U-bottom plates in RPMI 1640 medium supplemented with 10% fetal calf serum and 100 IU/mL penicillin and streptomycin at 37 °C with 5% CO_2_. T cells were stimulated with anti-CD3/CD28 activation (final concentrations of 2 μg/mL and 1 μg/mL, respectively) and cultured for 5 days. The cultural medium was replaced on the third day. The cells acquired from the proliferation of CD3+ T cells were analyzed by measuring CFSE intensity by flow cytometry *via* FACS Aria™ II flow cytometer (BD Biosciences, San Jose, CA, USA), and the data were analyzed using FlowJo 10.1 (FLowJo LLC, BD Biosciences, San Jose, CA, USA).

### 2.7 Statistical Analysis

Data were analyzed using the GraphPad Prism 8.0 software and expressed in median and range. The between-group difference was detected using the Mann–Whitney U nonparametric test, and the correlation between two concerning variables was evaluated using the Spearman rank correlation test. All statistical analyses were conducted using the SPSS version 19.0 software. A two-sided P value < 0.05 was considered significant.

## 3 Results

### 3.1 Demographic and Clinical Characteristics of the Patients

The demographic and clinical features of the 22 patients with MAP, 25 patients with SAP, and 26 HCs are presented in **Table 1**. No significant difference in the distributions of age or sex was observed in the study groups (p > 0.05). The numbers of white blood cells, neutrophils, and monocytes were increased in the patients with AP compared with the HCs. However, the lymphocytes decreased in the patients with AP, especially those with SAP (p<0.05) (**Table 1**). The AMY, CRP levels, and APACHE II scores of the patients with SAP were elevated compared with those with MAP and HCs (p<0.05). An increase in AP severity also corresponded to an extended length of hospitalized stay (LOS) (p<0.05).

**Table 1:** Demographic and characteristic of participants.

Parameters	HC	MAP	SAP
Number	26	22	25
Age (year range)	39 (21–64)	38 (15-63)	37 (21-60)
Gender (male/female)	13/13	17/5^*^	12/13
WBC (10^9^/L)	6.81 (4.7-9.42)	8.72 (4.82-17.31)^*^	9.62 (4.63-20.51)^*^
Neutrophils (10^9^/L)	3.82 (2.94-4.79)	6.13 (4.82-13.11)^*^	8.44 (3.99-18.24)^*^
Lymphocytes (10^9^/L)	2.53 (1.02-3.57)	1.94 (0.81-3.49)^*^	1.02 (0.4-2.31)^*^
Monocytes (10^9^/L)	0.45 (0.19-0.75)	0.64 (0.22-1.08)^*^	0.49 (0.20-1.75)
AMY (U/L)	32 (6-154)	284 (23-696)^*^	889 (84-2900)^*^
Ca (mmol/L)	2.41 (2.25-2.58)	2.18 (1.31-2.38)^*^	1.68 (0.84-2.34)^*^
LOS (d)	NA	7 (3-10)	16 (4-70)
CRP (g/L)	2.5 (0.6-7.21)	3.92 (0.91-193.4)^*^	212 (3.9-359)^*^
APACHE II score	NA	1 (0-5)	10 (5-19)

Data shown are median (range) or number of cases. HC, healthy control; MAP, mild acute pancreatitis; SAP, severe acute pancreatitis; WBC, white blood cell count; AMY, amylase; CRP, C-reactive protein; APACHE, acute physiology and chronic health evaluation; NA, not available; LOS, length of hospital stay; normal values: WBC: 4.0–10.0 (10^9^/L), neutrophils: 2.0–7.0 (10^9^/L), lymphocytes: 0.8–4.0 (10^9^/L), monocytes: 0.12–0.80 (10^9^/L), AMY: 8–220 (U/L), Ca 2.25–2.67(mmol/L), and CRP: 0–10 (mg/L). *p<0.05 compared with the HCs.

### 3.2 The Number of MDSC in Patients With AP Increased Compared With That in HCs

**Figure 1:**
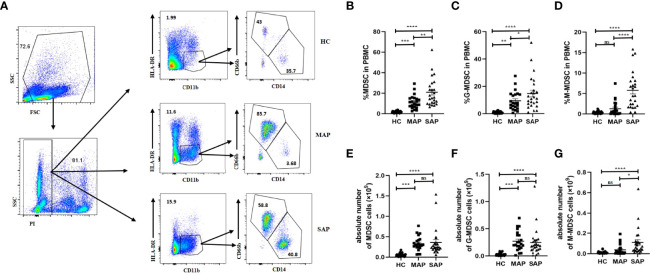
The percentage and absolute number of MDSC in PBMC in patients with AP was increased compared that in HCs. **(A)** The flow cytometry profiling of HLA-DR+CD11b+ MDSCs, and its subsets in PBMC in HC, MAP, and SAP groups. Percentages of MDSC **(B)**, G-MDSC **(C)**, M-MDSC **(D)** and absolute number of MDSC **(E)** and its subgroups **(F, G)** are higher in patients with MAP and SAP. *p < 0.05; **p < 0.01; ***p < 0.001; ****p < 0.0001; ns, not significant.

The proportion of HLA-DR^-^CD11b^+^ MDSC in the PBMCs of the SAP group (20.90 ± 13.580%) was significantly higher than that of the MAP group (11.40 ± 6.698%) and HC group (1.74 ± 0.9780%) (P<0.01). A significant difference in the proportion of MDSC was also observed between the patients with MAP and HCs (P =0.0009) (**Figures 1A** **, B**). In the subgroup analysis, the proportion of HLA-DR^-^CD11b^+^CD66b^+^CD14^-^ G-MDSC in the PBMCs of the SAP group (14.72 ± 12.190) was significantly higher than that of the HC group (0.9009 ± 0.7723%) and MAP group (9.477 ± 6.934%) (**Figure 1C**). The proportion of HLA-DR^-^CD11b^+^ CD66b^-^CD14^+^ M-MDSC in PBMCs was significantly higher in the SAP group than that in the HC and MAP groups (5.782 ± 4.486% vs. 0.3490 ± 0.3067%) (**Figure 1D**). Moreover, similar results were found for the cell count of MDSCs and their subsets. The absolute number of MDSCs, G-MDSCs, and M-MDSCs, in the AP group was higher than that in HC group (**Figures 1E** **–G**).

### 3.3 The Frequency of MDSCs Was Reduced After Treatment in the AP Group

**Figure 2:**
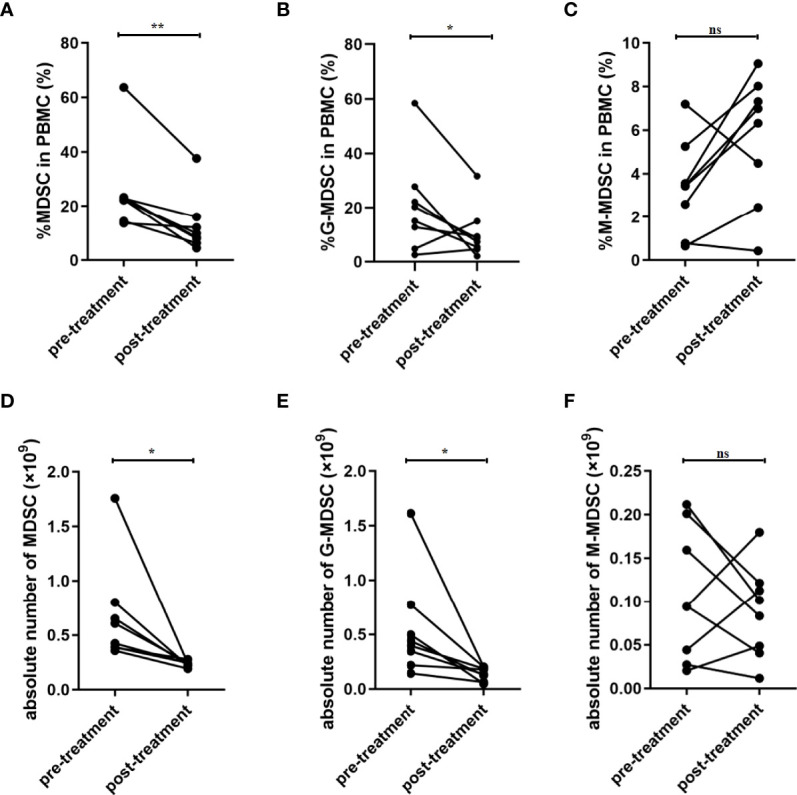
Frequency of MDSCs was decreased after treatment in patients with SAP. **(A)** Percentage of MDSC in PBMC was decreased after treatment. Percentage of G-MDSC **(B)** and M-MDSC **(C)** in PBMC had no significant difference after treatment. The absolute number of MDSC **(D)** and G-MDSC **(E)** was decreased after treatment. **(F)** The absolute number of M-MDSCs had no significant difference after treatment. n = 8. PBMC, peripheral blood mononuclear cell. *p <0.05; **p <0.01; ns, not significant.

Overall, eight patients were followed up after treatment. The biochemical data was summarized as **Table 2**. The median of APAHCE II scores were 10 and 3 before and after treatment, respectively (p=0.0002, **Table 2**). The mean CRP level before treatment was 191.9 ng/ml and 16.39 ng/ml after treatment (p=0.0003, **Table 2**). The percentage of MDSCs in PBMCs and the absolute number of MDSC after treatment significantly decreased compared to that before treatment (p<0.05, **Figures 2A and D**). The percentage and absolute number of G-MDSCs were reduced after treatment (p<0.05, **Figures 2B and E**). No significant differences were observed in the frequency and absolute number of M-MDSCs before and after treatment (**Figures 2C, F**).

**Table 2:** Effect of treatment on the values of clinical measures in follow-up AP patients.

Parameters	Before treatment	After treatment
Age (year range)	40.5 (23-60)	
Gender (male/female)	5/3	
WBC (10^9^/L)	11.07 (4.63-20.50)	8.77 (5.80-11.50)
Neutrophils (10^9^/L)	9.33 (3.99-18.24)	5.52 (4.01-8.40)
Lymphocytes (10^9^/L)	1.14 (0.40-1.75)	2.55 (1.16-4.51)^*^
Monocytes (10^9^/L)	0.52 (0.20-1.05)	0.71 (0.47-1.14) ^*^
AMY (U/L)	748 (84-2900)	71 (23-116) ^*^
CRP (g/L)	191.9 (76-306.1)	16.39 (3.9-32) ^*^
APACHE II score	10 (6, 17)	3 (2, 6) ^*^

Data are presented as median and range.

*p < 0.05 vs. values before treatment.

### 3.4 The Percentage of MDSCs in PBMCs Was Positively Correlated With AP

**Figure 3:**
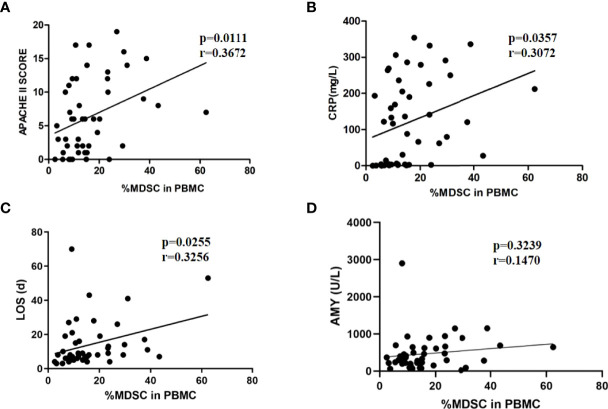
The positive correlation between MDSC frequency and severity of AP. MDSC frequency was positively correlated to APACHE II scores **(A)**, CRP levels **(B)**, and LOS **(C)**. **(D)** MDSC frequency had no relationship with amylase levels.

To confirm whether MDSCs were involved in the inflammatory response and disease progression of AP, the correlation between MDSCs and the severity of disease was analyzed. The APACHE II score, CRP level, and LOS were used to predict severity of AP in multiple researches (15, 16). The proportion of MDSCs in PBMCs was positively correlated with the APACHE II score (r=0.3672, p=0.0111) and CRP levels (r=0.3072, p=0.0357) in patients with AP (**Figures 3A** **–C**). Additionally, in the correlation analysis, the proportion of MDSCs in PBMCs was positively correlated with LOS (r= 0.3072, p=0.0255). The findings indicated that AMY level was elevated in the patients with AP, especially those with SAP (**Table 1**). However, the proportion of MDSCs in PBMCs had no statistical correlation with the AMY level (**Figure 3D**).

### 3.5 M-MDSC in PBMC Was Positively Correlated With AP Severity

**Figure 4:**
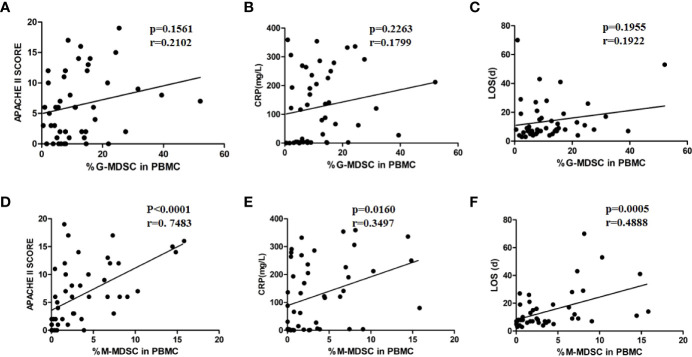
M-MDSC frequency was associated with disease severity. The percentage of G-MDSC in PBMC was not correlated with APCHE II scores **(A)**, CRP levels **(B)**, or LOS **(C)**. The percentage of M-MDSC in PBMC was positively related to APACHE II scores **(D)**, CRP levels **(E)**, and LOS **(F)**.

In the correlation analysis between the proportion of G-MDSCs and M-MDSCs in PBMCs and disease severity, the proportion of M-MDSCs in PBMCs was positively correlated with APACHE II scores, CRP levels, and LOS (**Figures 4D** **–F**). No correlation was found between the G-MDSC percentage in PBMCs and disease severity (**Figures 4A** **–C**).

### 3.6 The Proportion of MDSCs in Peritoneal Effusion of Patients With SAP Was Higher Than That in PBMCs

**Figure 5:**
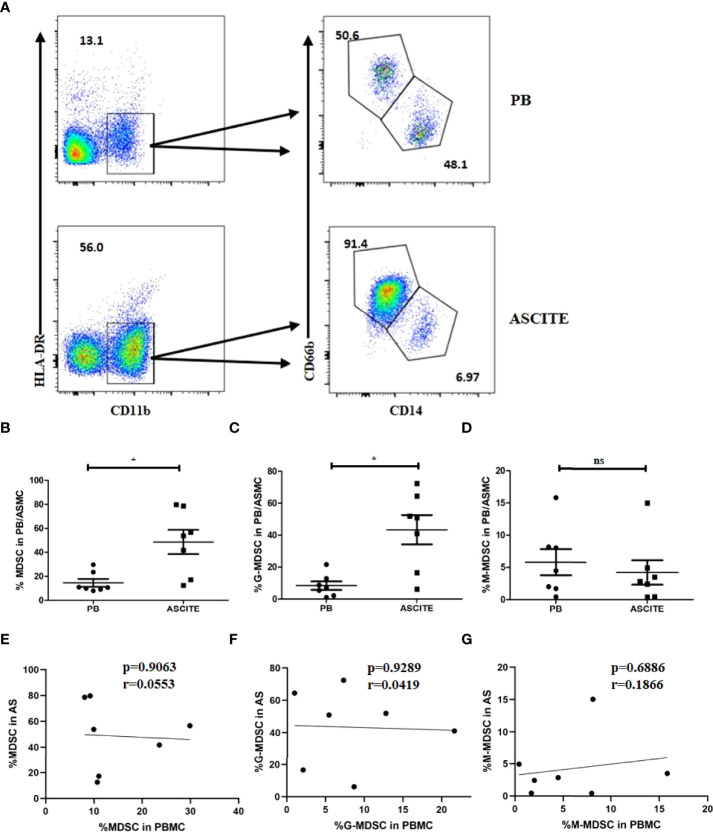
The percentage of MDSCs in ASMCs in patients with SAP. **(A)** The flow cytometry analysis of MDSC and its subsets in ASMC and peripheral PBMC in patients with SAP. Percentages of MDSC **(B)**, G-MDSC **(C)**, and M-MDSC **(D)** in ASMC and PBMC in SAP patients. No significant difference was observed between the percentages of MDSC **(E)**, G-MDSC **(F)**, and M-MDSC **(G)** in PBMC and in ASMC. n = 7, *p < 0.05; ns, not significant. ASMC, ascitic fluid mononuclear cell. PBMC, peripheral blood mononuclear cell.

The formation of local or peripancreatic effusion is one of the important indicators for SAP. Peripancreatic fluid contains biochemical substances, such as AMY, lipase, cytokines and chemokines, as well as immune cells. To confirm the specific function of MDSCs in the abdominal cavity, we collected a total of 7 ascite samples from patients with SAP who had abdominal puncture and drainage, and detected the proportion of MDSC and its subgroups component in ASMC. The proportion of MDSCs in ASMCs (48.66 ± 26.80%) was significantly higher than that in PBMCs (14.59 ± 8.494%, p = 0.0197, **Figures 5A, B**). For the subgroup analysis, the dominant group in ASMC was G-MDSC (43.37 ± 24.25%, **Figure 5A**). Proportion of G-MDSC in ascites was higher than that in the peripheral blood (**Figure 5C**). However, no significant difference was observed in the proportion of M-MDSCs in ASMCs and PBMCs (p = 0.5512, **Figure 5D**). To confirm the relationship of MDSCs between the peripheral blood and ascites, we compared the percentage of MDSCs in PBMCs and ASMCs. The frequency of MDSCs in ASMCs was not related to the frequency in PBMCs (**Figure 5E**). No significant difference was observed between G-MDSCs and M-MDSCs in the peripheral blood and ascites (**Figures 5F, G**).

### 3.7 MDSCs From Patients With SAP had Enhanced Inhibitory Function Compared With Those in HCs

**Figure 6:**
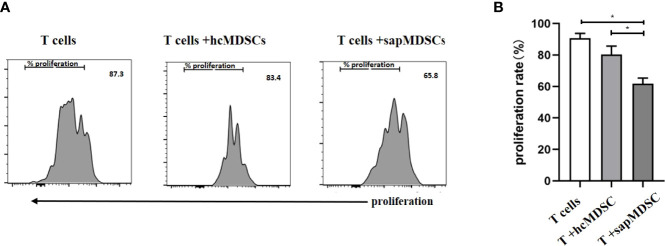
The inhibition of MDSC in peripheral blood from HCs and patients with SAP. **(A, B)** MDSCs from patients with SAP had increased inhibitory ability to CD3+ T cell proliferation compared to HCs. hcMDSCs, MDSC from healthy control; sapMDSC, MDSC from patients with SAP. CD3+ T cells were cultured or co-cultured with MDSC from HC or SAP patients for 5 days, and CD3+T cells proliferated. * P < 0.05. The experiment was repeated three times, and the data were expressed in the form of (mean + SD). The Student’s t test was used for statistical analysis.

MDSCs inhibit T cell proliferation or T cell function, such as cytokine secretion. The isolated CD3+ T cells were cultured *in vitro* for 5 days with or without MDSCs. The ratio between CD3+ T cells and MDSCs was 2:1. The results indicated that the proliferation rate of CD3+ T cell in the HC group had no significant difference compared with the T cell group (p = 0.1201). However, the proliferation rate of CD3+ T cells in patients with SAP (61.77 ± 2.076%) was lower than that in the HC group (80.33 ± 3.117%) as well as the CD3+ T cell group (**Figures 6A and B**).

### 3.8 MDSCs From Patients With SAP had Increased Levels of Arg-1 and ROS

**Figure 7:**
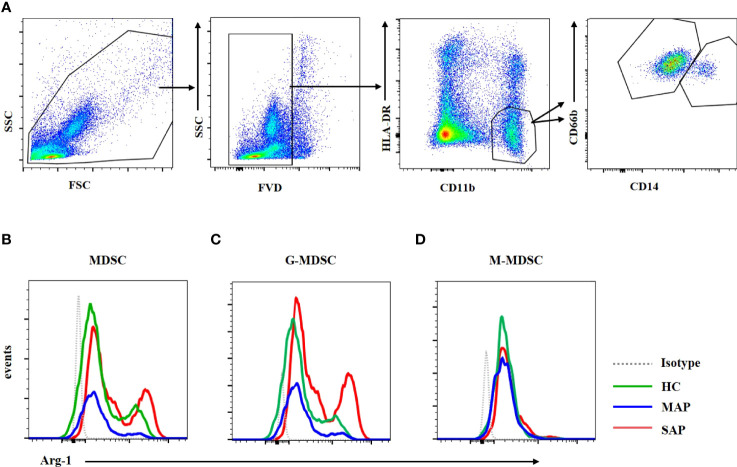
Arg-1 expression in MDSC and G-MDSC was increased in patients with SAP and MAP compared with HCs. **(A)** The flow cytometry profiling of HLA-DR+CD11b+ MDSCs, CD66b+CD14- G-MDSC and CD66b-CD14+ M-MDSC. Arg-1 expression of MDSC **(B)** and G-MDSC **(C)** was increased in patients with MAP and SAP. **(D)** Arg-1 expression in M-MDSC subset from HC, MAP, and SAP had no difference.

**Figure 8:**
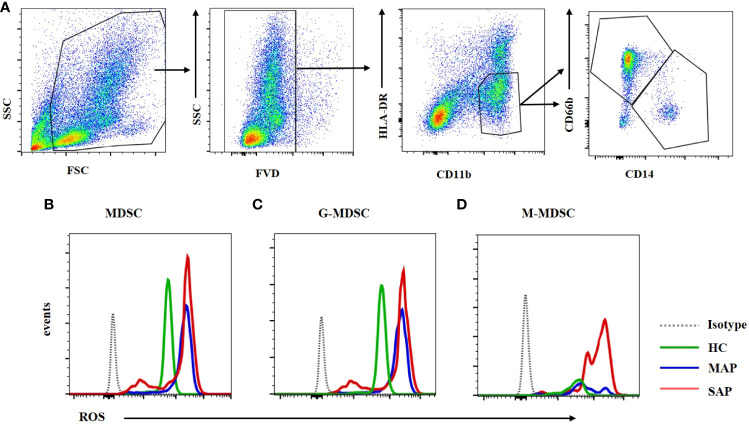
ROS expression in MDSC was enhanced in the MAP and SAP patients compared with HCs. **(A)** The flow cytometry profiling of HLA-DR+CD11b+ MDSCs, CD66b+CD14- G-MDSC and CD66b-CD14+ M-MDSC. ROS expression of MDSC **(B)** G-MDSC **(C)** and M-MDSC **(D)** was increased in patients with MAP and SAP compared with HCs.

MDSCs play their inhibitory function by Arg-1, iNOS, TGF-β, IL-10, COX2, indoleamine 2,3-dioxygenase (IDO) sequestration of cysteine (17). Arg-1 which is located in the cytoplasm is detected by intracellular staining *via* FCM. Dot plots of MDSC and its subsets profiling ware shown on **Figure 7A**. The expression of Arg-1 in the peripheral blood MDSC and G-MDSC from patients with SAP was significantly higher than that of HCs (**Figures 7B** **, C**). The Arg-1 expression in M-MDSCs from HCs and patients with MAP and SAP had no significant difference (**Figure 7D**). ROS could be detected *via* FCM after transmembrane dye staining. MDSC and its subsets analyzed for ROS detection was shown on **Figure 8A**. The ROS expression of the MDSCs in the patients with AP, especially those with SAP, was remarkably increased (**Figure 8B**), including the G-MDSC and M-MDSC subsets (**Figures 8C, D**).

## 4 Discussion

In this study, the proportion and quantity of MDSCs in PBMC in the peripheral blood of the patients with AP, especially those with SAP, were significantly increased compared with those in the HCs. Furthermore, the proportion of MDSCs in PBMCs was positively correlated with the APACHE II score, CRP level, and LOS, which were considered as the presentation of disease severity in AP. After treatment, the frequency of MDSCs was decreased remarkably. To date, no study has been conducted on the MDSC frequency in patients with AP. In an AP mouse model, the proportion and number of CD11b^High^GR-1^High^ cells in pancreatic tissues were significantly higher than that in the unmodeled group, suggesting that MDSC was involved in local pancreatic inflammation in the mouse AP model (18). In the case of bacterial inflammatory diseases, MDSC frequency in the peripheral blood of patients suffering from sepsis and in sepsis mouse models was significantly increased, which was positively correlated with disease severity. The MDSCs lasted long in patients with septic shock (19). These studies were consistent with our experimental results. Moreover, the subgroup analysis revealed that G-MDSCs and M-MDSCs were higher in patients with AP than those in HCs, and G-MDSCs were the most dominant. Hence, G-MDSC may be a neutrophil precursor or a low-density neutrophil. Neutrophils are involved in the local and inflammatory reaction of AP, which is consistent with the increase of neutrophils in the peripheral blood during AP (20). M-MDSC is considered a precursor of DCs or macrophages. Here, the M-MDSCs were significantly increased in the peripheral blood of the patients with AP. This result was consistent with that of the increase in mononuclear macrophages in the peripheral blood (21). Our study indicated that the proportion of MDSCs and its subgroup in PBMCs can be used as a predictor of AP severity.

Peritoneal effusion is formed because of higher portal pressure, visceral artery dilation, or decreased plasma colloid osmotic pressure in patients with AP. Furthermore, peritoneal effusion is localized around the pancreas and/or accumulated extensively in the abdominal cavity according to the severity of the disease. In our analysis of MDSCs and their subset proportion in ASMCs, the proportion of MDSCs in the peritoneal effusion was higher than that in the PBMCs of the same patient.

To the best of our knowledge, no study exists concerning MDSCs and their subset proportion in peritoneal effusion of patients with AP. However, studies on mononuclear cells in peritoneal effusion of patients with liver cirrhosis and cancer have revealed that MDSCs were higher in patients with liver cancer (22), which suggested that increased MDSCs in the abdominal cavity may contribute to the pathophysiology of the disease. The MDSC subset analysis indicated that G-MDSCs were the dominant subgroup, and the proportion of G-MDSC was remarkably increased compared with that of peripheral blood. This result was consistent with the increased number of neutrophils in the peripheral blood as well as in the pancreatic tissues in AP (23). Neutrophils are considered as the initial group of immune cells that are recruited by necrotic acinar cells and amplify inflammatory response during the pathophysiology of AP. The accumulation of immature neutrophils in the peritoneal effusion facilitates local pancreatic inflammation formation. However, as a side effect, the extensive immunosuppression of cells could lead to an abdominal secondary infection. In our study, the sample number in each group was insufficient to analyze the differences between infection and non-infection cases. More studies are needed to confirm the function of localized MDSCs in disease progression or development of secondary infection in AP.

We performed T cell proliferation test to analyze the inhibitory functional of MDSCs from HC and AP patients. Our study showed that MDSCs derived from SAP patients had increased inhibitory function to CD3+ T cell proliferation. Arg-1 and ROS expressions of MDSCs are considered to be the main inhibitory factors of MDSC (24). Arginase suppresses T cell function by depleting arginine from the microenvironment. The increased activity of arginase in MDSCs contributes to enhanced L-arginine catabolism, which consumes the non-essential amino acid from the microenvironment. The shortage of L-arginine inhibits T-cell proliferation *via* deducing their CD3ζ expression (25). ROS inhibit T cells through inhibition of DNA synthesis and alterations in T-cell receptor signaling (26). An elevated ROS expression has been confirmed as one of the main characteristics of MDSCs in tumor-bearing mice as well as in patients with cancer (27, 28). Blocking ROS in MDSCs isolated from mice and patients with cancer completely abolished the suppressive effect of these cells *in vitro* (29, 30). A study has demonstrated that in patients with sepsis, early expansion of ARG1-producing G-MDSCs correlated with increased T cell dysfunction and increased susceptibility to secondary infections in the surgical intensive care unit (31). Sunitinib, the tyrosine kinase inhibitors, elevated activity and proliferation of CD8+ cells by reducing STAT3 activation and ARG1 expression which decreased the circulating MDSC number (32). In our study, the Arg-1 and ROS expressions were both enhanced in the SAP group. SAP was pancreatic and systemic infection caused by waterfall inflammatory response triggered by damage and necrosis of pancreatic acinar cells. It facilitates to control over-reacting inflammation that involves SIRS, which is directly related with organ damage. However, overimmunosuppression induces CARS, contributing to secondary infection in the clinical progression. In this study, the expansion of MDSCs and increased suppressive function of them contributed CARS reaction. MDSC and its subsets played an important role in keeping the balance of SIRS and CARS. As the increased inhibitory function of MDSCs in acute pancreatitis, adaptive MDSCs transferring strategies may restrain the process of AP, but the feasibility needs animal study to confirm.

## Conclusion

MDSCs, an immune-impressive subset of immune cells, were involved in immune tolerance in the clinical process of AP. The increased levels of MDSCs were correlated with poor clinical outcomes of patients with AP. The percentage of MDSCs in PBMCs was a readily applicable biomarker for AP severity. Moreover, the immunosuppressive function of MDSCs in T cell proliferation was increased in the peripheral blood of patients with SAP, which is associated with the high level of Arg-1 and ROS expression. More investigations are needed in the future to confirm the actual function of MDSCs in AP, providing clinical application of MDSCs as a therapeutic target for AP, especially in SAP.

## Data Availability Statement

The original contributions presented in the study are included in the article/supplementary material. Further inquiries can be directed to the corresponding author.

## Ethics Statement

The studies involving human participants were reviewed and approved by the Human Ethics Committee of Jilin University (approval number: 2019-323). The patients/participants provided their written informed consent to participate in this study.

## Author Contribution

LD: Data curation(lead), writing-original draft. MW and DW: design. HC and HW: data analysis. PG: Conceptualization. All authors contributed to the article and approved the submitted version.

## Funding

This work is supported by the grant from the National Natural Science Foundation of China (82071853), the Science and Technology Department of Jilin Province (20190201140JC), the National Natural Science Foundation of Jilin Province (JLSCZD2019-008), the National Science and Technology Major Project (2018ZX10302206, 2018ZX10723203).

## Conflict of Interest

The authors declare that the research was conducted in the absence of any commercial or financial relationships that could be construed as a potential conflict of interest.

## Publisher’s Note

All claims expressed in this article are solely those of the authors and do not necessarily represent those of their affiliated organizations, or those of the publisher, the editors and the reviewers. Any product that may be evaluated in this article, or claim that may be made by its manufacturer, is not guaranteed or endorsed by the publisher.

## Abbreviations

AMY, amylase; AP, acute pancreatitis; Arg, arginase; ASMCs, ascitic fluid mononuclear cells; CARS, compensatory anti-inflammatory syndrome; CFSE, carboxyfluorescein succinimidylester; FVD, Fixable Viability Dye; G-MDSCs, granulocytic MDSCs; HC, healthy control; LOS, length of stay; M-MDSCs, monocytic MDSCs; MAP, mild acute pancreatitis; MDSCs, myeloid-derived suppressor cells; PBMCs, peripheral blood mononuclear cells; PI, propidium iodide; ROS, reactive oxygen species; SAP, severe acute pancreatitis; SIRS, systemic inflammatory responses syndrome.
